# Application Value of In-House HIV-1 DNA Genotypic Resistance Testing for Individuals with Low-Level Viremia

**DOI:** 10.3390/v18101098

**Published:** 2026-10-04

**Authors:** Ying Zhou, Chuanfeng Zhang, Qi Sun, Tao Qiu, Qianjin Fan, Jing Lu, Haiyang Hu, Xiaoqin Xu, Dandan Yang, Haitao Yang

**Affiliations:** Department of HIV/STD Control and Prevention, Jiangsu Provincial Center for Disease Control and Prevention, Nanjing 210009, China; zhouy@jscdc.cn (Y.Z.); zhangchuanfeng@jscdc.cn (C.Z.); sq_njmu@163.com (Q.S.); qiutao@jscdc.cn (T.Q.); fanqianjin@jscdc.cn (Q.F.); lujing@jscdc.cn (J.L.); huhaiyang@jscdc.cn (H.H.); xqxu18@sina.com (X.X.); yangdan1997@163.com (D.Y.)

**Keywords:** HIV-1, DNA genotypic drug resistance testing, evolutionary divergence time

## Abstract

Background: To provide evidence for HIV-1 DNA genotypic resistance testing (GRT) strategies in low-level viremia (LLV) populations by comparing the amplification efficiency and resistance mutation profiles between HIV-1 DNA GRT and RNA GRT. (2) Methods: Convenience sampling was used for participant enrolment. Plasma HIV-1 RNA and peripheral blood monocyte (PBMC) DNA were extracted separately. The protease–reverse transcriptase (PR-RT) region was amplified via an in-house protocol, followed by Sanger and Nanopore sequencing. The exact McNemar’s test and Fisher’s test were applied to compare amplification success rates. Univariate and multivariable logistic regression analyses implemented in R were conducted to identify factors associated with the detection of HIV-1 drug-resistant strains. BEAST-based molecular clock analysis was performed to calculate the divergence time between RNA-derived and DNA-derived sequences. (3) Results: A total of 209 samples were enrolled, and valid sequences were obtained from 166 samples. Within the viral load range of 50–200 copies/mL, HIV-1 DNA GRT yielded significantly higher amplification success rates than RNA GRT (*p* < 0.05). The overall resistance detection rate for DNA GRT (20.8%) was lower than that for RNA GRT (33.7%), with discrepancies mainly involving nucleoside reverse transcriptase inhibitor (NRTI)- and non-nucleoside reverse transcriptase inhibitor (NNRTI)-associated mutations. Individuals receiving antiretroviral therapy (ART) for 7–18 months exhibited higher odds of detecting drug-resistant strains using either DNA GRT or RNA GRT. The median evolutionary divergence time between matched RNA- and DNA-derived sequences was 3.13 years. By contrast, the median evolutionary divergence time between paired DNA generated by Sanger and Nanopore sequencing was 0.84 years. (4) Conclusions: DNA GRT serves as a valuable supplementary screening tool in public health scenarios of LLV with failed RNA GRT amplification. However, clinical interpretation should combine RNA GRT findings, longitudinal viral load monitoring and treatment history, rather than relying solely on DNA GRT to modify antiretroviral regimens.

## 1. Introduction

Suppressive combination antiretroviral therapy (ART) has diminished mortality and morbidity for people living with HIV-1 (PLWH) due to its highly efficient viral suppression [1]. The development of HIV-1 drug resistance mutations (DRMs) remains a persistent barrier to sustained HIV viral load (VL) suppression in PLWH receiving ART [2]. Several studies have linked LLV to the emergence of multi-drug resistance (MDR) mutations [3,4]. Nevertheless, some studies have reported no association between very low-level viremia (LLV) (20–50 copies/mL) or LLV with negative immunological and virological consequences among ART-treated individuals [5]. More recently, DNA GRT has been suggested as an alternative to the standard RNA GRT method in the absence of viremia and/or states of low viremia (<200 copies/mL plasma) [6,7]. Evidence describing the dynamic behavior of the HIV latent reservoir demonstrates that any viral variant, including drug-resistant ones, can persist within reservoirs and highlights the advantages of DNA GRT [8,9]. However, it remains unclear whether the DNA reservoirs harboring DRMs can be stably replicated, packaged into viral particles, and release into the peripheral blood. Furthermore, clinical guidance for DNA GRT is limited by insufficient empirical evidence. To provide evidence for DNA GRT’s application value for LLV, we compared the assay performance and resistance profiles between DNA GRT and RNA GRT.

## 2. Materials and Methods

### 2.1. Ethics

In China, PLWH receiving ART are entitled to free annual viral load (VL) testing according to the ‘Free National AIDS Antiviral Drug Treatment Handbook’. The World Health Organization (WHO) defines LLV as a viral load of 50–1000 copies/mL, and we follow the WHO definition for LLV [10]. This study was granted approval by the Ethics Committee of the Jiangsu Provincial Center for Disease Control and Prevention (JSJK2026-B024-01). The study was conducted in compliance with the Declaration of Helsinki. Written informed consent was obtained from all participants at the initiation of antiretroviral therapy.

### 2.2. Participants and Sampling

Samples analyzed in our study were convenience specimens collected for routine drug resistance testing among individuals with LLV. Individuals with VLs of 50–1000 copies/mL and age ≥ 18 years were involved. Both plasma and peripheral blood monocytes (PBMCs) were isolated from each blood sample after 3000 r/min centrifugation and stored at −80 °C prior to nucleic acid extraction.

### 2.3. Laboratory Detection

HIV-1 RNA and total DNA extraction and amplification were performed simultaneously on a single sample. HIV-1 RNA was extracted from 500 uL plasma using the Livzon nucleic acid extraction kit (Livzon, Zhuhai, China). Total DNA was extracted from 200 ul of PBMCs using the QIAsymphony DNA Mini Kit (QIAGEN, Hilden, Germany). The partial pol sequence that corresponded to codons 1 to 99 of the protease and codons 1–299 of the reverse transcriptase was amplified by in-house nested reverse transcription PCR. For RNA first-round amplification, the primers MAW26 (5′-TTGGAAATGTGGAAAGGAAGGAC-3′) and RT-21 (5′CTGTATTTCTGCTATTAAGTCTTTTGATGGG-3′) were used with a Vazyme one-step PCR kit (Vazyme Biotechnology Company, Nanjing, China). For DNA first-round amplification, the same primers were used with a Biotech 2*Taq PCR mix kit (Sangon Biotech Company, Shanghai, China). Both the RNA and DNA reaction systems were 50 μL, and 10 ul of template was added for each reaction. The primers PRO-1 (5′-CAGAGCCAACAGCCCCACCA-3′) and RT-20 (5′-CTGCCAGTTCTAGCTCTGCTTC-3′) were used for PR-RT with a Biotech 2*Taq PCR mix kit (Sangon Biotech Company, Shanghai, China) for both RNA and DNA amplification [11]. The reaction volume for the second-round PCR was 50 ul for both the RNA and DNA templates, with 10 μL of template added for each reaction. The amplification products were sent to a company for Sanger sequencing and Nanopore sequencing (Beijing Bomaide Gene Technology Co., Ltd., Beijing, China). Nanopore sequencing can test base site frequencies at/above a 10% threshold wtih PromethION 2 Solo (Oxford Nanopore Technologies), improving the sensitivity for the detection of mutation sites. All sequences were submitted to an HIV drug resistance database (https://hivdb.stanford.edu/, accessed on 12 July 2026) to determine the DRMs and resistance levels to protease inhibitors (PIs), nucleoside reverse transcriptase inhibitors (NRTIs), and non-nucleoside reverse transcriptase inhibitors (NNRTIs). There are five DR levels, namely susceptible, potential low-level resistance, low-level resistance, mid-level resistance, and high-level resistance.

### 2.4. Statistical Analysis

An exact McNemar’s test was used to compare the amplification success rates between RNA GRT and DNA GRT. Univariate and multivariable logistic regression analyses were implemented in R to identify factors associated with the detection of HIV-1 drug-resistant strains. *p* < 0.05 was considered as statistically significant.

### 2.5. Sequence Evolutionary Analyses

Molecular clock analysis was used to evaluate the divergence time between DNA GRT-derived and RNA GRT-derived sequences. Sequences simultaneously undergoing RNA GRT with Sanger sequencing and DNA GRT with Sanger and Nanopore sequencing were involved in sequence evolutionary analyses. The Bayesian Markov chain Monte Carlo (MCMC) method was applied in Beast X v10.5.0. We used the uncorrelated relaxed molecular clock with a gamma-distributed general time reversible substitution model. We chose the GRT nucleotide substitution model. The Bayesian SkyGrid coalescent model was used. A chain length of 200 million MCMC generations was applied. Evolutionary parameters were sampled every 2000 generations. The convergence of the MCMC output log was assessed in Tracer v1.7.2, and all parameters achieved effective sample sizes (ESSs) > 200. Removing 10% of burn-in and combining evolutionary parameters, clock trees were generated using TreeAnnotator v10.5.0. Itol (https://itol.embl.de/, accessed on 7 August 2026), which was used to display the evolutionary tree.

## 3. Results

### 3.1. Number of Samples and Sequences

**Figure 1:**
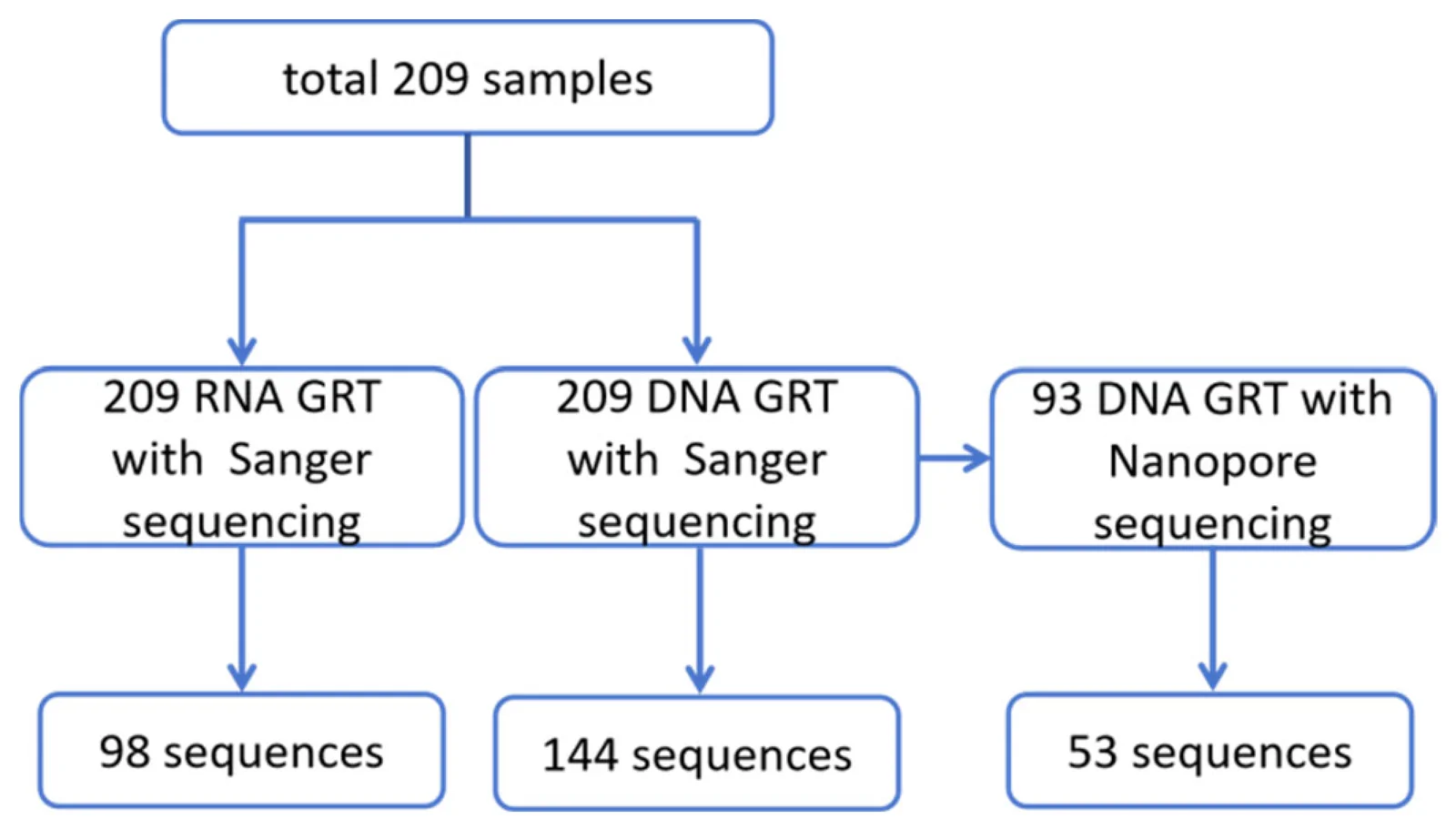
The sampling course of GRT.

In total, 209 samples were enrolled; 166 samples were amplified successfully, obtaining a total of 242 sequences. Of these, 98 sequences were amplified from RNA GRT and 144 sequences from DNA GRT. We selected 93 samples randomly from these 209 samples for additional Nanopore sequencing and obtained 53 sequences (Figure 1). The dominant HIV-1 subtypes among the 166 samples were CRF07_BC (37.3%, 62/166) and CRF01_AE (31.9%, 53/166), followed by CRF0107 (7.8%, 13/166), CRF08_BC (9%, 15/166), B (3.6%, 6/166), and URF (3.6%, 6/166). The remaining samples (6.6%, 11/166) comprised subtypes C, CRF48/67/68_01B, CRF85_BC, and CRF87_cpx.

### 3.2. Comparison of RNA and DNA GRT Capabilities

The detection capabilities of DNA GRT were compared against those of RNA GRT in different ranges of VLs. The results demonstrated that DNA GRT presented significantly higher amplification success within VL ranges of 50–100 copies/mL and 101–200 copies/mL (*p* < 0.05). However, no statistically significant differences in amplification success were observed in other ranges of VLs above 200 copies/mL (Table 1).

### 3.3. Differences in Resistance Profiles Between RNA and DNA GRT

**Figure 2:**
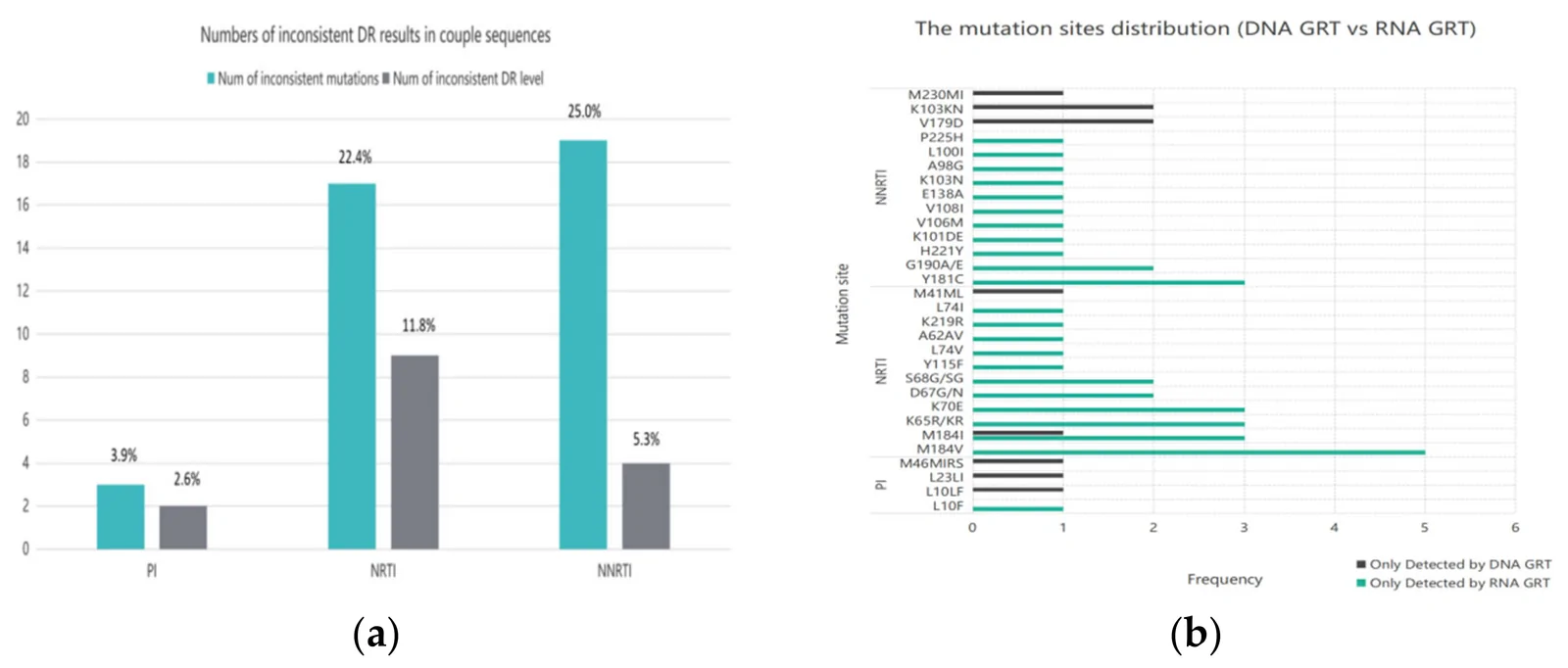
(**a**) The number and rate of inconsistent DR results in coupled sequences. (**b**) The inconsistent DRM distributions detected by DNA and RNA GRT.

The overall drug resistance rates were 20.8% as detected by DNA GRT and 33.7% as detected by RNA GRT. The most discrepancies were localized to NRTI- and NNRTI-associated resistance (Table 2). DNA GRT- and RNA GRT-derived sequences from a single sample were analyzed to reveal the mutation detection differences in detail. Paired sequence comparisons identified discordant mutation sites in 76 matched sequence pairs. Sequences differing in mutation sites, including degeneracy mutation sites, were defined as inconsistent sequences. Sequences differing in DR levels were defined as inconsistent DR level sequences. We found three pairs (3.9%) that were inconsistent in mutation sites in PI, 17 pairs (22.4%) inconsistent in NRTI, and 19 pairs (25.0%) inconsistent in NNRTI. The sequence numbers of inconsistent DR levels caused by differences in mutation were two pairs in PI, nine pairs in NRTI, and four pairs in NNRTI. The highest rate of inconsistent mutations was 25% in NNRTI, whereas the highest rate of inconsistent DR levels was 11.8% in NRTI (Figure 2a). Mutation sites detected by these two methods were also compared with sequences in pairs. DNA GRT detected mutation sites M46, L23, and L10 in PI, which RNA GRT did not detect, while RNA GRT only detected L10, which DNA GRT did not detect in mutation sites from inconsistent sequences. In NRTI and NNRTI, RNA GRT detected 11 mutation sites that were not detected by DNA GRT, while DNA GRT detected two and three mutation sites that were not detected by RNA GRT in mutation sites from inconsistent sequences (Figure 2b and Table S1).

### 3.4. Correlated Factor Analysis of Resistance Detection Capabilities Between RNA GRT and DNA GRT

Candidate predictive variables included sex, age at diagnosis, age at sampling, transmission route, CD4 count, time from diagnosis to sampling, interval between diagnosis and ART initiation, time receiving ART, and ART regimen. The results showed that only the number of months receiving ART exhibited a significant difference. The odds ratios (*ORs*) of the factors of 7–12 months and 13–18 months in DNA GRT were both 23. The corresponding *OR* values for RNA GRT were 27.50 (7–12 months) and 66.00 (13–18 months). These illustrate that individuals receiving ART for 7–18 months exhibit substantially elevated odds of drug-resistant strain detection by both DNA GRT and RNA GRT. No other analyzed variables demonstrated significant associations (Table 3).

### 3.5. Divergence Time Between DNA and RNA GRT Sequences

**Figure 3:**
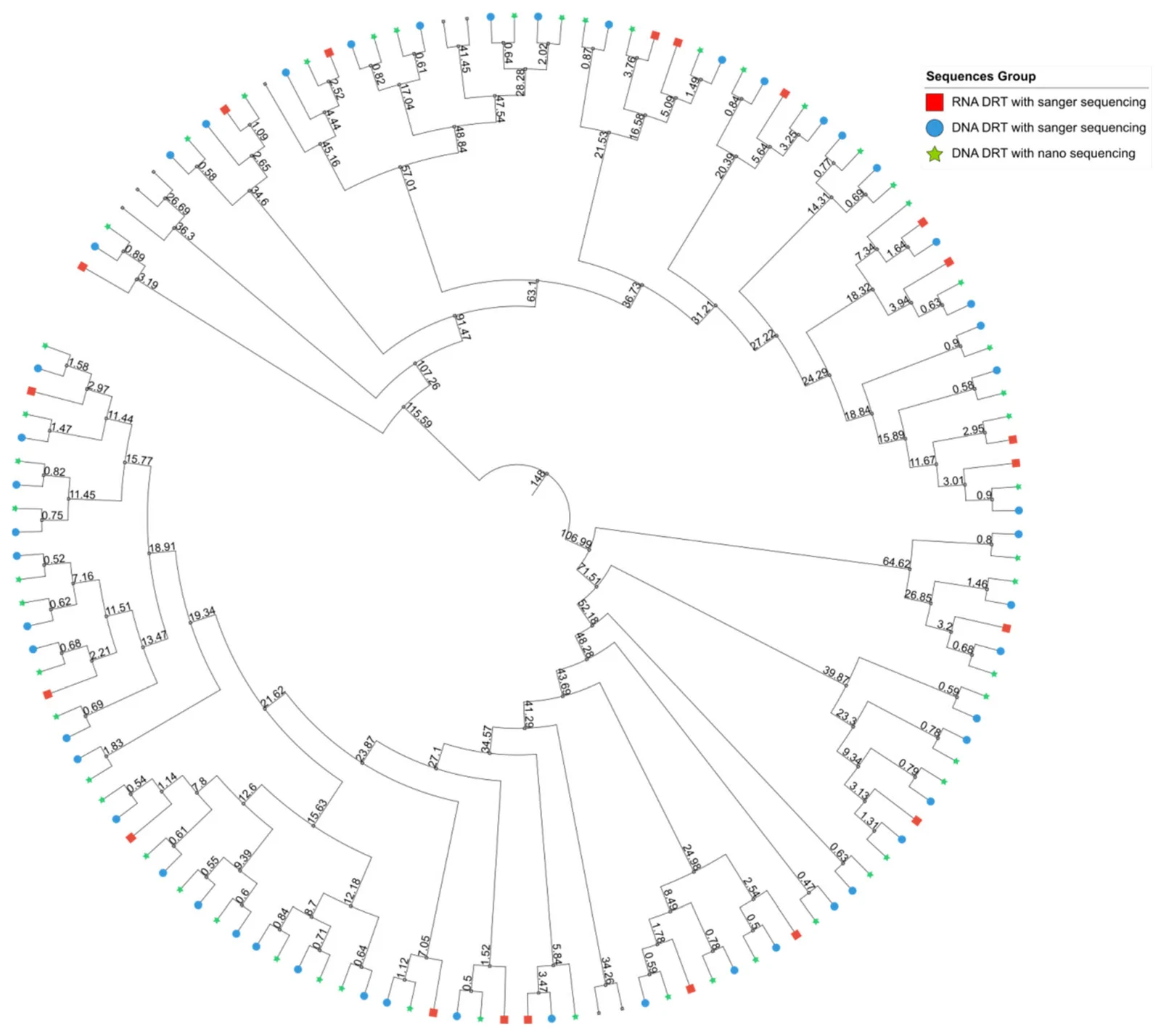
Molecular clock tree constructed with sequences detected by three-platform sequencing from a single sample.

Among the 93 samples detected simultaneously with RNA GRT undergoing Sanger sequencing (RNAS) and DNA GRT undergoing Sanger (DNAS) and Nanopore sequencing (DNAN), we obtained 36 sequences from RNA GRT and 70 sequences from DNA GRT with Sanger sequencing, as well as 53 sequences from DNA GRT with Nanopore sequencing. After this, complete triplet datasets (RNAS + DNAS + DNAN) were available for 18 samples. On the molecular clock tree, sequences originating from the same participant clustered within terminal clades. Each node displayed a divergent age between sequences (Figure 3). Sequence sets were classified into three categories: (1) triplets of RNAS, DNAS, and DNAN sequences for one sample; (2) paired RNAS and DNAN sequences; (3) paired DNAS and DNAN sequences. In category 1, 15 out of 18 RNAS sequences diverged earlier than their paired DNAS counterparts. The sequence divergence time range of RNAS and DNAS spans 1.14–7.05 years, with a median value of 3.13 years. The sequence divergence time range of DNAS and DNAN spans 0.50–3.25 years, with a median value of 0.84 years. In category 2, two sequence pairs yielded divergence times of 2.96 years and 3.76 years. In category 3, the sequence divergence time range of DNAS and DNAN spans 0.47 years to 2.02 years, with a median value of 0.75 years.

## 4. Discussion

This study evaluated the application value of in-house HIV-1 DNA genotypic drug resistance testing for individuals with LLV. Through the pairwise comparison of the amplification success rates, drug resistance mutation profiles, resistance detection correlates, and evolutionary divergence times between DNA GRT and RNA GRT, we confirm that HIV-1 DNA GRT possesses unique detection advantages in the LLV population (VLs ≤ 200 copies/mL). Nevertheless, certain discrepancies existed in the drug resistance results yielded by DNA GRT and RNA GRT, and neither method can fully substitute the other.

In the viral load range of 50–200 copies/mL, DNA GRT achieved significantly higher amplification success rates than RNA GRT. Under LLV conditions, the plasma HIV-1 RNA template abundance is extremely low, which frequently leads to amplification failure. This constitutes a major reason for the frequent absence of valid results from RNA GRT in LLV individuals in clinical practice [12]. By contrast, HIV-1 DNA within PBMCs represents viral forms integrated into the host cell genome in the viral reservoir and is not directly governed by the contemporaneous plasma viral load. Even when the plasma viral RNA levels are very low, stable viral DNA templates persist in host cells [13,14]. Therefore, DNA GRT can compensate for the shortcomings of RNA GRT, which often fails at very low viral loads; this is consistent with findings from several previous studies [15]. When the viral load exceeds 200 copies/mL, the amplification success rates are comparable for both approaches, indicating that conventional RNA GRT remains the preferred assay for individuals with high-level viremia (HLV) with sufficient plasma RNA templates.

In our study, the overall drug resistance rate of DNA GRT (20.8%) was lower than that of RNA GRT (33.7%), and the same discrepancies in resistance outcomes were predominantly observed in NRTI- and NNRTI-associated mutation sites. Meanwhile, the drug resistance rate obtained in Asia among individuals with LLV was reported to be as high as 40.7% in other studies—higher than the resistance rate in our study [16,17]. In individuals with lower levels of virus replication, it may take several months for plasma virus DRMs to reach detectable levels in PBMCs. This time lag can explain why, in individuals with LLV, PBMC GRT is more sensitive than the historical plasma virus RNA GRT in the detection of DRMs [7]. The HIV-1 DNA reservoir harbors viral sequences accumulated through multiple prior rounds of viral replication, encompassing both dominant viral strains currently undergoing active replication and historical viral strains suppressed by antiretroviral drugs that no longer produce progeny virions. Plasma RNA, however, mainly reflects circulating viral populations under the current drug selection pressure and more directly represents contemporary dominant variants at present [18]. On one hand, certain resistance mutations detected exclusively by RNA GRT may be attributable to the transient presence of replication-competent variants carrying such mutations in the plasma. These viruses are not substantially integrated into host cellular genomes, rendering them undetectable by DNA GRT owing to the low template abundance of this viral strain. On the other hand, DNA GRT recovered PI-associated mutations (M46, L23, L10) that were absent in the paired RNA GRT data, suggesting that cellular reservoirs preserve mutated viral variants that have disappeared from the plasma [19,20]. These mutations represent archived historical variants and may not correspond to the phenotypic profile of the currently actively replicating virus.

Given the observed discrepancy in the resistance detection rates, we explored predictors of resistance variant detection. Several studies have reported factors associated with LLV-associated resistance [21]. Meanwhile, our regression analyses indicated that most factors considered in this study were not significantly related to the resistance detection outcomes. Only the duration of ART exposure showed robust statistical associations for both DNA GRT and RNA GRT. Participants receiving ART for 7–18 months had markedly elevated ORs of detecting drug-resistant strains. A limited number of factors were associated with the detection of drug resistance, reflecting the complexity underlying resistance emergence among individuals with LLV. Furthermore, the results for DNA GRT and RNA GRT were consistent in the analysis of predictors.

Molecular clock evolutionary analyses further illuminated the genetic evolutionary disparities between DNA-derived and RNA-derived sequences. Sequences from RNAS, DNAS, and DNAN derived from identical samples clustered at terminal branches of the phylogenetic tree, confirming their shared evolutionary origin. The median divergence time between RNAS and DNAS sequences was 3.13 years, indicating that contemporary circulating plasma virus populations are evolutionarily separated from prior archived proviral templates by multiple years. Proviral DNA reservoirs record viral genetic information dating back years, which does not fully equate to contemporaneous plasma viral populations. Longitudinal analyses have also found that resistance-associated mutations (RAMs) in plasma HIV-1 emerge more than a year before being found in PBMCs [22]. These findings partially support the time results derived from our evolutionary analysis. Furthermore, our results indicate that plasma HIV-1 RNA sequences are derived from earlier viral variants, whereas DNA sequences originate from recently evolved viral populations. This finding suggests that HIV-1 DNA undergoes continuous turnover and evolution within host cells; however, the sequences capable of expressing and producing HIV-1 virions are mostly derived from proviruses integrated into the human genome years ago. It seems that early-integrated HIV-1 DNA possesses a more stable replicative capacity. In comparison, the median divergence time between paired DNAS and DNAN datasets from identical samples was only 0.84 years—substantially lower than the divergence between RNA and DNA sequences. This finding suggests that sequences from DNAS and DNAN mainly stemmed from biological homology. Although Nanopore sequencing enables the detection of low-frequency mutations above a 10% threshold and can capture a broader reservoir quasi-species diversity, platform-specific advantages cannot overcome the intrinsic evolutionary divergence between archived provirus and contemporary plasma virus populations.

This study has several limitations. First, convenience sampling was adopted instead of consecutive enrolment, which may have introduced selection bias. Second, only the PR-RT region was targeted in this study, whereas other gene segments, such as the integrase region, were not included. Third, RNA amplification has a low success rate, affecting the usable data profiles.

## 5. Conclusions

Our results demonstrate that DNA GRT serves as a valuable supplementary screening tool in public health scenarios of LLV when RNA GRT amplification fails. Resistance mutations identified via DNA GRT may represent archived historical variants and do not necessarily drive contemporary treatment failure. Therefore, the cautious interpretation of DNA GRT reports is warranted in clinical practice. Additionally, a negative DNA GRT result cannot definitively rule out contemporaneous circulating resistance mutations at the plasma RNA level. Therefore, the optimal clinical strategy should integrate results from both RNA GRT and DNA GRT, together with dynamic viral load monitoring and prior antiretroviral treatment histories, for comprehensive clinical judgement.

## Figures and Tables

**Table 1:** The detection capabilities of DNA GRT and RNA GRT.

VL Interval (Copies/mL)	Number of Samples	Rate of Sequences Obtained from RNA GRT (%)	Number of Sequences Obtained from RNA GRT Only	Rate of Sequences Obtained from DNA GRT (%)	Number of Sequences Obtained from DNA GRT Only	*p*
50–100	92	32.6	10	62.2	38	*p* < 0.05
101–200	59	37.3	3	71.2	23	*p* < 0.05
201–400	19	63.2	4	63.2	4	1
401–600	12	75.0	4	58.3	2	0.68
601–1000	27	92.6	1	92.6	1	0.28

**Table 2:** The resistance rates detected by DNA GRT and RNA GRT.

	DR Rate			PI		NRTI		NNRTI	
	Num of Resistance Sequences	Num of All	DR Rate	Num of Resistance Sequences	DR Rate	Num of Resistance Sequences	DR Rate	Num of Resistance Sequences	DR Rate
DNA	30	144	20.8%	5	3.5%	11	7.6%	28	19.4%
RNA	33	98	33.7%	4	4.1%	19	19.4%	29	29.6%

Num = Number.

**Table 3:** (**a**) Correlates of DNA GRT detection. (**b**) Correlates of RNA GRT detection.

(a)									
DNA GRT									
Variable		*p*	OR	95% CI		*P1*	OR	95% CI	
Sex									
	Male								
	Female	0.70	0.77	0.21	2.88				
Age at diagnosis		0.07	0.98	0.95	1.00				
Age at sampling		0.10	0.98	0.95	1.00				
Transmission route		0.83							
	Heterosexual transmission								
	Homosexual transmission	0.55	1.31	0.54	3.17				
	Venous blood draw	1.00	0.00	0.00					
	Unknown	0.52	2.28	0.19	27.93				
	Other	0.30	4.56	0.26	79.88				
CD4 count		0.39							
	0–200								
	201–350	0.39	0.61	0.20	1.88				
	351–500	0.82	0.88	0.29	2.64				
	>500	0.10	0.38	0.12	1.21				
Time from diagnosis to sampling (years)		0.56							
	0–2								
	3–5	0.33	0.54	0.16	1.84				
	6–10	0.98	1.01	0.37	2.74				
	>10	0.45	1.61	0.47	5.47				
Interval between diagnosis and ART initiation		0.13							
	0								
	1–3	0.10	0.27	0.06	1.26				
	4–12	0.23	2.67	0.55	13.02				
	13–24	0.54	0.51	0.06	4.40				
	>24	0.19	2.13	0.68	6.67				
Time receiving ART (months)		0.02				0.01			
	0–6								
	7–12	0.01	20.13	2.13	189.88	0.01	23.00	2.40	220.34
	13–18	0.01	23.00	2.02	262.56	0.01	23.00	2.02	262.56
	19–24	0.29	3.83	0.32	46.69	0.29	3.83	0.32	46.69
	>24	0.10	5.75	0.72	45.83	0.10	5.66	0.71	45.28
ART regimen		0.72							
	BIC/EVG + FTC + TAF								
	TDF + 3TC + EFV/RPV/ANV	0.37	0.62	0.22	1.77				
	TDF + 3TC + LPVr	1.00	0.00	0.00					
	3TC + DTG + LPVr/TDF/ABC	0.65	1.35	0.38	4.84				
	AZT + 3TC + EFV/NVP	0.96	0.97	0.24	3.95				
**(b)**									
**RNA GRT**									
**Variable**		***p***	**OR**	**95% CI**		***P1***	**OR**	**95% CI**	
Sex									
	Male								
	Female	0.53	0.64	0.16	2.56				
Age at diagnosis		0.18	0.98	0.95	1.01				
Age at sampling		0.10	0.98	0.95	1.01				
Transmission route		0.87							
	Heterosexual transmission								
	Homosexual transmission	0.66	1.23	0.49	3.07				
	Venous blood draw	/	/	/	/				
	Unknown	0.55	2.40	0.14	42.26				
	Other	0.55	2.40	0.14	42.26				
CD4 count		0.46							
	0–200								
	201–350	0.37	0.57	0.17	1.93				
	351–500	0.62	0.73	0.21	2.53				
	>500	0.12	0.41	0.13	1.26				
Time from diagnosis to sampling (years)		0.60							
	0–2								
	3–5	0.30	0.56	0.18	1.68				
	6–10	0.61	0.76	0.26	2.18				
	>10	0.26	0.43	0.10	1.85				
Interval between diagnosis and ART initiation		0.20							
	0								
	1–3	0.32	0.56	0.18	1.75				
	4–12	0.82	1.20	0.26	5.63				
	13–24	0.42	0.40	0.04	3.70				
	>24	0.07	4.00	0.89	17.99				
Time receiving ART (months)		0.01				0.01			
	0–6								
	7–12	0.01	27.50	2.00	378.84	0.01	27.50	2.00	378.84
	13–18	0.01	66.00	3.47	1254.57	0.01	66.00	3.47	1254.57
	19–24	0.33	3.67	0.27	49.29	0.33	3.67	0.27	49.29
	>24	0.17	4.40	0.53	36.61	0.18	4.35	0.52	36.33
ART regimen		0.17							
	BIC/EVG + FTC + TAF								
	TDF + 3TC + EFV/RPV/ANV	0.10	0.39	0.13	1.18				
	TDF + 3TC + LPVr	/	/	/	/				
	3TC + DTG + LPVr/TDF/ABC	0.69	0.75	0.18	3.06				
	AZT + 3TC + EFV/NVP	0.06	0.18	0.03	1.07				

*p*: the difference value analyzed with single-factor logistic regression. *P1*: the difference value analyzed with multi-factor logistic regression.

## Data Availability

Data are contained within this article.

## Supplementary Materials

The following supporting information can be downloaded at https://www.mdpi.com/article/10.3390/v18101098/s1. Table S1: The DR and ART regimen details of 76 matched samples.

## Abbreviations

The following abbreviations are used in this manuscript:

GRT	Genotypic resistance testing
LLV	Low-level viremia
PR-RT	Protease–reverse transcriptase
ART	Antiretroviral therapy
PLWH	People living with HIV-1
DRMs	Drug resistance mutations
VL	Viral load
MDR	Multi-drug resistance
WHO	World Health Organization
PBMCs	Peripheral blood monocytes
PI	Protease inhibitor
NRTI	Nucleoside reverse transcriptase inhibitor
NNRTI	Non-nucleoside reverse transcriptase inhibitor
MCMC	Markov chain Monte Carlo
ESSs	Effective sample sizes
ORs	Odds ratios
RNAS	RNA GRT with Sanger sequencing
DNAS	DNA GRT with Sanger sequencing
DNAN	Nanopore sequencing
RAMs	Resistance-associated mutations
