# Supplementary material for: Application Value of In-House HIV-1 DNA Genotypic Resistance Testing for Individuals with Low-Level Viremia
Source: Viruses. 2026 Oct 4;18(10):1098. doi: 10.3390/v18101098 (PMC13709528; doi:10.3390/v18101098)
Supplement: Supplementary file 1 [file viruses-18-01098-s001.zip › viruses-4541687-supplementary.pdf]

Table S1. The DR and ART regimen details of 76 matched samples

Sequence Name	Drug resistance profiles	Drug resistance mutations	ART regimens
Sample1			3TC+EFV+TDF
Sample1PBMC			
Sample2	NRTI，NNRTI	K65R,D67G,V75M,Y115FK103N,Y188H,H221Y	3TC+EFV+TDF
Sample2PBMC	NRTI，NNRTI	K65KR,V75VIM,K103KN,Y188H	
Sample3			3TC/DTG
Sample3PBMC			
Sample4		Q58E	3TC+EFV+TDF
Sample4PBMC			
Sample5			3TC/DTG
Sample5PBMC			
Sample6			3TC+EFV+TDF
Sample6PBMC		M46MIRS,L23LI	
Sample7		V179D	ANV/3TC/TDF
Sample7PBMC		V179D	
Sample8			3TC+EFV+TDF
Sample8PBMC			
Sample9			3TC+EFV+TDF
Sample9PBMC			
Sample10			BIC/FTC/TAF
Sample10PBMC			
Sample11			3TC+EFV+TDF
Sample11PBMC			

Sequence Name	Drug resistance profiles	Drug resistance mutations	ART regimens
Sample12	NRTI, NNRTI	K65R, K101DE, V106M, Y181C, G190A	3TC+EFV+TDF
Sample12PBMC	NNRTI	K103KN	
Sample13			ANV/3TC/TDF
Sample13PBMC			
Sample14	PI	L10F	3TC/DTG
Sample14PBMC	None		
Sample15	NRTI, NNRTI	M184V, Y188L	3TC/DTG
Sample15PBMC	PI, NRTI, NNRTI	L10LF, M184MV, Y188YFHL	
Sample16			No information
Sample16PBMC			
Sample17			3TC+TDF+NVP
Sample17PBMC			
Sample18		V106I	EFV+3TC+AZT
Sample18PBMC	*NNRTI*	V106I, M230MI	
Sample19			ANV/3TC/TDF
Sample19PBMC			
Sample20	NRTI, NNRTI	D67N, K70E, Y115F, M184VK101E, V106M, G190A	3TC/DTG+TDF
Sample20PBMC	NRTI, NNRTI	D67DN, K70KE, Y115YF, M184MV, K101KE, V106VM, G190GA	
Sample21			3TC+EFV+TDF
Sample21PBMC			
Sample22			3TC+EFV+TDF
Sample22PBMC			
Sample23	NNRTI	K103N	3TC/DTG
Sample23PBMC			

Sequence Name	Drug resistance profiles	Drug resistance mutations	ART regimens
Sample24		V179E	3TC+EFV+TDF
Sample24PBMC		V179E	
Sample25	NNRTI	K103S,E138A	BIC/FTC/TAF
Sample25PBMC	NNRTI	K103S,E138A	
Sample26			3TC+NVP+TDF
Sample26PBMC			
Sample27	NRTI，NNRTI	M184VK103N,V108I	3TC+EFV+TDF
Sample27PBMC	NNRTI	K103N	
Sample28		Q58E	AZT/3TC+EFV
Sample28PBMC		Q58E	
Sample29			AZT+3TC+EFV
Sample29PBMC		V179D	
Sample30			3TC+EFV+TDF
Sample30PBMC			
Sample31		S68G	3TC+DTG+TDF
Sample31PBMC		S68G	
Sample32	NNRTI	V179D,Y188L	BIC/FTC/TAF
Sample32PBMC	NNRTI	V179D,Y188L	
Sample33		V179D	BIC/FTC/TAF
Sample33PBMC		V179VDIN	
Sample34		V179E	BIC/FTC/TAF
Sample34PBMC		V179E	
Sample35			3TC/DTG
Sample35PBMC			

Sequence Name	Drug resistance profiles	Drug resistance mutations	ART regimens
Sample36	*NRTI, NNRTI*	D67N, K70E, L74VE138A, G190E	3TC+EFV+TDF
Sample36PBMC	*None*		
Sample37	NRTI, NNRTI	A62AV, K65R, S68SG, M184I, K101E, V179T, Y181C, G190S	3TC+EFV+TDF
Sample37PBMC	NNRTI	K101KE, V179IT, Y181YC, G190GS	
Sample38			BIC/FTC/TAF
Sample38PBMC			
Sample39		V179E	ANV/3TC/TDF
Sample39PBMC		V179E	
Sample40			3TC+EFV+TDF
Sample40PBMC			
Sample41	NNRTI	K101E, V179T, G190S	3TC/DTG+LPV/r
Sample41PBMC	NNRTI	K101E, V179T, G190S	
Sample42			AZT/3TC+EFV
Sample42PBMC			
Sample43	NRTI, NNRTI	L74I, M184V, E138K, F227L	BIC/FTC/TAF
Sample43PBMC	NRTI, NNRTI	L74I, M184V, E138K, F227L	
Sample44	NRTI, NNRTI	A62V, K65R, M184I, Y181C, G190S	3TC+EFV+TDF
Sample44PBMC	NRTI, NNRTI	A62AV, K65KR, M184MI, Y181C, G190S	
Sample45			BIC/FTC/TAF
Sample45PBMC			
Sample46			3TC+RPV+TDF
Sample46PBMC			
Sample47			3TC+EFV+TDF
Sample47PBMC			

Sequence Name	Drug resistance profiles	Drug resistance mutations	ART regimens
Sample48			NVP+AZT/3TC
Sample48PBMC			
Sample49	NNRTI	K103N,E138A	NVP+AZT+3TC
Sample49PBMC	NRTI，NNRTI	M184I,E138A,V179D	
Sample50	NNRTI	K103N	3TC+EFV+TDF
Sample50PBMC	NNRTI	K103N	
Sample51	NRTI	M184V,V106I	No information
Sample51PBMC	None	V106I	
Sample52	NRTI，NNRTI	M184I,V179D,Y181C	BIC/FTC/TAF
Sample52PBMC	NRTI，NNRTI	M184MI,V179VD,Y181YC	
Sample53	*NRTI，NNRTI*	K70E,M184V,V179E,Y181C	BIC/FTC/TAF
Sample53PBMC	*None*	V179E	
Sample54	NNRTI	A98G,K103N	3TC+EFV+TDF
Sample54PBMC	NNRTI	A98G,K103N	
Sample55			3TC+EFV+TDF
Sample55PBMC			
Sample56	NRTI，NNRTI	M184I,K103N,Y181C	No information
Sample56PBMC	NNRTI	K103KN,Y181C	
Sample57			3TC+EFV+TDF
Sample57PBMC			
Sample58	NNRTI	K101E,V179T,G190S	DTG/ABC/3TC
Sample58PBMC	NNRTI	K101E,V179T,G190S	
Sample59	NNRTI	K101E,V179T,G190S	BIC/FTC/TAF
Sample59PBMC	NNRTI	K101E,V179T,G190S	

Sequence Name	Drug resistance profiles	Drug resistance mutations	ART regimens
Sample60			AZT/3TC+EFV
Sample60PBMC			
Sample61			3TC+EFV+TDF
Sample61PBMC			
Sample62			3TC+EFV+TDF
Sample62PBMC			
Sample63			3TC/DTG
Sample63PBMC			
Sample64			3TC/DTG
Sample64PBMC			
Sample65			3TC+AZT+EFV
Sample65PBMC			
Sample66	NRTI，NNRTI	K65R,S68N,Y115F,M184V,V106M,V179D,F227L	3TC+EFV+TDF
Sample66PBMC	NRTI，NNRTI	K65R,S68SN,Y115F,M184V,V106M,V179D,F227FL	
Sample67	*NRTI，NNRTI*	S68G,K70E,M184I,A98G,V179D,Y181C	BIC/FTC/TAF
Sample67PBMC	*None*	S68G,V179D	
Sample68	NRTI，NNRTI	L74I,M184V,K219R,E138K,F227L	BIC/FTC/TAF
Sample68PBMC	NRTI，NNRTI	L74I,K103KN,E138EK,F227FL	
Sample69			3TC+DTG+TDF
Sample69PBMC			
Sample70		E138A	BIC/FTC/TAF
Sample70PBMC		E138A	
Sample71			3TC+EFV+TDF
Sample71PBMC			

Sequence Name	Drug resistance profiles	Drug resistance mutations	ART regimens
Sample72			3TC+EFV+TDF
Sample72PBMC			
Sample73	NRTI，NNRTI	Q58E,K65KR,S68G,L74I,M184V,L100I,K103N,P225H	3TC+EFV+TDF
Sample73PBMC	NRTI，NNRTI	Q58E,M41ML,K103N	
Sample74			3TC+EFV+TDF
Sample74PBMC			
Sample75			3TC+EFV+TDF
Sample75PBMC			
Sample76			BIC/FTC/TAF
Sample76PBMC			

Background: Yellow- Inconsistent DR Level in PI; Green- Inconsistent DR level in NRTI.

Italic text: Inconsistent DR Level in NNRTI.

Blank cell：NO DRMS detected.

PBMC: DNA GRT result
